# Release of GLP-1 and PYY in response to the activation of G protein-coupled bile acid receptor TGR5 is mediated by Epac/PLC-ε pathway and modulated by endogenous H_2_S

**DOI:** 10.3389/fphys.2014.00420

**Published:** 2014-11-03

**Authors:** Vanitha Bala, Senthilkumar Rajagopal, Divya P. Kumar, Ancy D. Nalli, Sunila Mahavadi, Arun J. Sanyal, John R. Grider, Karnam S. Murthy

**Affiliations:** ^1^Gastroenterology Division, Department of Internal Medicine, Virginia Commonwealth UniversityRichmond, VA, USA; ^2^Department of Physiology, VCU Program in Enteric Neuromuscular Sciences, Virginia Commonwealth UniversityRichmond, VA, USA

**Keywords:** cystathionine-γ-lyase, GPCR signaling, oleanolic acid, enteroendocrine cells

## Abstract

Activation of plasma membrane TGR5 receptors in enteroendocrine cells by bile acids is known to regulate gastrointestinal secretion and motility and glucose homeostasis. The endocrine functions of the gut are modulated by microenvironment of the distal gut predominantly by sulfur-reducing bacteria of the microbiota that produce H_2_S. However, the mechanisms involved in the release of peptide hormones, GLP-1 and PYY in response to TGR5 activation by bile acids and the effect of H_2_S on bile acid-induced release of GLP-1 and PYY are unclear. In the present study, we have identified the signaling pathways activated by the bile acid receptor TGR5 to mediate GLP-1 and PYY release and the mechanism of inhibition of their release by H_2_S in enteroendocrine cells. The TGR5 ligand oleanolic acid (OA) stimulated Gα_s_ and cAMP formation, and caused GLP-1 and PYY release. OA-induced cAMP formation and peptide release were blocked by TGR5 siRNA. OA also caused an increase in PI hydrolysis and intracellular Ca^2+^. Increase in PI hydrolysis was abolished in cells transfected with PLC-ε siRNA. 8-pCPT-2′-*O*-Me-cAMP, a selective activator of Epac, stimulated PI hydrolysis, and GLP-1 and PYY release. L-Cysteine, which activates endogenous H_2_S producing enzymes cystathionine-γ-lyase and cystathionine-β-synthase, and NaHS and GYY4137, which generate H_2_S, inhibited PI hydrolysis and GLP-1 and PYY release in response to OA or 8-pCPT-2′-*O*-Me-cAMP. Propargylglycine, an inhibitor of CSE, reversed the effect of L-cysteine on PI hydrolysis and GLP-1 and PYY release. We conclude: (i) activation of Gα_s_-coupled TGR5 receptors causes stimulation of PI hydrolysis, and release of GLP-1 and PYY via a PKA-independent, cAMP-dependent mechanism involving Epac/PLC-ε/Ca^2+^ pathway, and (ii) H_2_S has potent inhibitory effects on GLP-1 and PYY release in response to TGR5 activation, and the mechanism involves inhibition of PLC-ε/Ca^2+^ pathway.

## Introduction

Hormones released from the gastrointestinal tract play an important role in the regulation of several physiological functions including food intake, gastrointestinal motility and secretions, glucose and lipid metabolism, and energy expenditure (Sarah et al., 2005; Grudell and Camilleri, 2007; Holst, 2007). Glucagon-like peptide-1 (GLP-1) and peptide YY (PYY) are secreted from open-type neuroendocrine epithelial cells (L-cells) located in the distal ileum and colon that come in direct contact with gut nutrients (Eissele et al., 1992; Holzer et al., 2012).

GLP-1, a potent glucose-dependent insulin-stimulating hormone (MacDonald et al., 2002), also stimulates β cell proliferation and pro-insulin gene expression (Buteau et al., 2003), and inhibits glucagon expression (Mojsov, 2000). The synthesis of GLP-1 from pro-glucagon is directed by specific expression of convertase 1/3 in L-cells; cleavage of pro-glucagon by convertase 1/3 yields equimolar amounts of GLP-1 and GLP-2, together with the glucagon-containing peptides, glicentin and oxyntomodulin (Holst, 2007). The primary stimulus of GLP-1 secretion is the presence of nutrients, mainly fat and bile salts, in the distal ileum. Although both glucose and partly digested proteins can stimulate GLP-1 release when added directly to the distal ileum, both nutrients are predominantly absorbed by the proximal intestine. An early rise in the circulating GLP-1 has been attributed to GIP, a distinct glucose-dependent insulin-stimulating hormone released by glucose from the proximal intestine. The plasma enzyme dipeptidyl peptidase-IV inactivates circulating GLP-1 making its half-life short (less than 2 min) (Pospisilik et al., 2002; Holst, 2007).

PYY, a potent appetite regulating hormone that belongs to the neuropeptide Y and pancreatic polypeptide family of peptides, is co-localized with GLP-1 in the L-cells (Holzer et al., 2012). PYY content increases from proximal to distal regions in the GI tract. The two endogenous forms of PYY released into the circulation are PYY1-36 and PYY3-36; the latter is from the cleavage of tyrosine-proline residues from the N-terminal end of PYY1-36 and is the predominant form of PYY in the L-cells and in the circulation (Holzer et al., 2012). The stimulus for PYY release is luminal nutrients including glucose, protein and lipid digestion products, and bile salts (Reimann et al., 2004; Jahan-Mihan et al., 2011; De Silva and Bloom, 2012).

The role of altered levels of GLP-1 and PYY in the pathogenesis of diseases such as diabetes, obesity and non-alcoholic fatty liver diseases has been suggested and understanding the mechanisms of their release and regulation, might greatly contribute to the development of therapeutics (Valassi et al., 2009). Studies in cultures of mixed intestinal epithelial cells and in model enteroendocrine cells (e.g., STC-1 and GLUTag) have demonstrated the expression of the bile acid G protein-coupled receptor, TGR5, and the ability of bile acids to activate TGR5 and stimulate GLP-1 secretion (Kidd et al., 2008; Parker et al., 2012). TGR5-mediated stimulation of GLP-1 secretion was dependent on cAMP formation and was blocked by inhibitors of adenylyl cyclase (Katsuma et al., 2005; Parker et al., 2012).

H_2_S, produced as a byproduct by luminal sulfate-reducing commensal bacteria in the colon or as an endogenous signaling molecule synthesized from L-cysteine mainly via cystathionine-γ-lyase (CSE) and cystathionine-β-synthase (CBS), and to lesser extent by 3-mercaptopyruvate sulfurtransferase (3-MST), regulate various physiological functions such as secretion, motility and visceral nociception (Robert et al., 2003; Distrutti et al., 2006; Wallace, 2010; Li et al., 2011; Wang, 2012). The role of H_2_S in the regulation of GLP-1 and PYY release is not known. Therefore, the aims of this study were (i) to determine the mechanism of GLP-1 and PYY release by TGR5 activation, and (ii) to determine the effect of endogenous H_2_S on TGR5-mediated GLP-1 and PYY release. We show that cAMP-dependent GLP-1 and PYY release in response to TGR5 activation reflects activation of Epac (an exchange protein activated directly by cAMP) and PLC-ε, and release of Ca^2+^. We also show that stimulation of endogenous H_2_S formation by activation of CSE inhibited TGR5-dependent GLP-1 and PYY release by blockade of the Epac/PLC-ε pathway.

## Materials and methods

### Materials

Oleanolic acid was obtained from Sigma Aldrich (St. Louis, MO). TGR5 antibody was obtained from Abcam (Boston, MA). [^35^S]GTPγS, [^125^I]cAMP, [^3^H]myo-inositol were obtained from NEN Life Sciences Products (Boston, MA). U73122 and myristoylated PKI were obtained from Calbiochem (La Jolla, CA). Rabbit polyclonal antibody to TGR5 and PLC-ε were purchased from Abcam (Cambridge, MA), antibodies to Gα_s_, Gα_q_, Gα_i1_, Gα_i2_, and Gα_i3_ were from Santa Cruz Biotechnology Inc. (Santa Cruz, CA). Lipofectamine™ 2000 transfection reagents and SuperScript™ II Reverse Transcriptase kit were obtained from Invitrogen (Carlsbad, CA). All other reagents were obtained from Sigma (St. Louis, MO).

### Cell culture

Murine enteroendocrine cells (STC-1) were obtained from American Type Culture Collection (ATCC) and cultured in Dulbecco's Modified Eagle's Medium (DMEM) containing 10% fetal bovine serum (FBS) with 2 mM L-glutamine. Cells were maintained at 37°C in a humidified atmosphere of 5% CO_2_/95% air.

### Detection of TGR5, CBS, and CSE in STC-1 cells by RT-PCR

STC-1 cells were treated with the RNAqueous reagent (Ambion, Austin, TX) for total RNA extraction. The potentially contaminated genomic DNA was removed by treating 10 μg of the RNA sample at 37°C for 30 min with 1 μl of TURBO DNase (Ambion, Austin, TX) followed by an extraction with phenol:chloroform:isoamylalcohol (25:24:1). RNA (2 μg) was used to synthesize cDNA using SuperScript II reverse transcriptase (Applied Biosystems, Foster, CA) with random hexanucleotide primers. Conventional PCR was performed on cDNA using the HotMaster Taq DNA polymerase kit (Epicentre Biotechnologies, Madison, WI). The primers for mouse TGR5 (GenBank Accession No.NM_174985.1) were as follows; Forward 5′-CCC ACC GCC AGC TGT GTG AG-3′ and Reverse 5′-CCC CAT GGC CAC AGG CAC AG-3′, generating a fragment of 269 bp. The primers for mouse CBS (GenBank Accession No. NM_144855.2) were as follows; Forward 5′-GGT GGT GGC GTC TGC GTG TT-3′ and Reverse 5′-AGG CCT GGT CTC GTG ATT GGA TCT G-3′, generating a fragment of 345 bp. The primers for mouse CSE (GenBank Accession No. AY262829.1) were as follows; Forward 5′-GGG CAT CTG CAG GGA AAG GAA CG-3′ and Reverse 5′-GCA GAT TGG TCC ACG CCC CT-3′, generating a fragment of 851 bp.

### Determination of TGR5 and PLC-ε by western blot

Lysates were prepared from STC-1 cells and were separated by SDS-PAGE followed by transfer onto nitrocellulose membranes (Immobilon-FL, Millipore, Billerica, MA). The membranes were blocked, incubated with antibodies to TGR5 (1:1000) or PLC-ε (1:1500) (Abcam, Cambridge, MA; Proteintech, Chicago, IL), and following washing, incubated with secondary antibody conjugated to horseradish peroxidase (Santa Cruz biotechnology, Santa Cruz, CA). Proteins on the membrane were detected by the enhanced chemiluminescence detection system (Amersham Life Science Buckinghamshire, UK).

### Identification of TGR5 receptor-activated G proteins in STC-1 cells

G proteins selectively activated by the TGR5 ligand were identified as described previously (Okamoto et al., 1991; Murthy and Makhlouf, 1996). Ten ml of cells suspension (2 × 10^6^ cells/ml) were homogenized in 20 mM HEPES medium (pH 7.4) containing 2 mM MgCl_2_, 1 mM EDTA, and 2 mM dithiothreitol. After centrifugation at 27,000 × *g* for 15 min, the crude membranes were incubated for 30 min at 37°C with 100 nM [^35^S]GTPγS in a solution containing 10 mM HEPES (pH 7.4), 100 μM EDTA, and 10 mM MgCl_2_. The reaction was stopped with 10 volumes of 100 mM Tris-HCl medium (pH 8.0) containing 10 mM MgCl_2_, 100 mM NaCl, and 20 μM GTP and the mixture was placed in wells pre-coated with specific G protein antibodies (1:1000 dilution). Coating with G protein antibodies (1:1000) was done after the wells were first coated with anti-rabbit IgG for 2 h on ice. After incubation for 2 h on ice, the wells were washed three times with phosphate buffer solution containing 0.05% Tween 20, and the radioactivity from each cell was counted by liquid scintillation.

### Ca^2+^ release

Cells were plated in Matrigel-coated dishes (MatTek, Ashland, MA) 1–3 days prior to use and loaded with fura-2 AM by incubation for 2 h in a medium containing 25 mM Hepes (pH 7.4), 120 mM NaCl, 4.0 mM KCl, 1 mM CaCl_2_, and 1 mM MgCl_2_, and 14 mM glucose at room temperature. Cells were then washed, and dishes mounted on an inverted fluorescence microscope (Olympus IX71, Southall, UK) with a 40 × oil-immersion objective. Excitation at 340 and 380 nm was achieved using a 75 W Xenon arc lamp with a monochromator (Cairn Research, Faversham, UK) controlled by MetaFluor software (Universal Imaging; Cairn Research) and emission was recorded with a CCD camera (Orca ER, Hammamatsu; Cairn Research). Background-subtracted fluorescence was normalized to a baseline average measured before application of the first test reagent and expressed as a 340/380 nm ratio, and the response to OA was defined as increase in 340/380 ratio.

### cAMP assay

OA (10 μM) was added to 0.5 ml of cell suspension (10^6^ cells/ml) in the presence of 3-isobutyl-1-methylxanthine (IBMX, 10 μM), and the reaction was terminated after 60 s with 6% cold trichloroacetic acid (Vol/Vol). The mixture was centrifuged at 2000 × *g* for 15 min at 4°C; the supernatant was extracted three times with 2 ml diethyl ether, and the samples were lyophilized. The samples were reconstituted and cAMP was measured in triplicate using 100 μl aliquots by radioimmunoassay as described before (Murthy and Makhlouf, 1995). Results are expressed as pmol/mg protein.

### Transfection of siRNA

siRNA oligonucleotides specific for mouse TGR5 and PLC-ε were obtained from Invitrogen (Carlsbad, CA). The sequence of TGR5 siRNA was as follows; Forward 5′-CCC AAC UUC UCC UUC CUC UTT-3′ and Reverse TGR5: 5′-AGA GGA AGG AGA AGU UGG GTT-3′. Double-stranded RNA oligoribonucleotide NNGCGCGCUUUGUAGGAUUCA (5′-3′) was used as a control siRNA. The sequence of PLC-ε siRNA was as follows; Sense: 5′ CUG AUC CUC AAG ACG UUA Att 3′ and Antisense: 5′ UUA ACG UCU UGA GGA UCA Gtt 3′. STC-1 cells were seeded on 100 mm plates and allowed to adhere overnight. At the time of transfection, cells were 80–85% confluent and the siRNAs were transfected into STC-1 cells at a final concentration of 20 μM by use of lipofectamine 2000 reagent (Invitrogen Corp., Carlsbad, CA) according to the manufacturer's instructions. Cells were kept for 48 h before use for biochemical measurements.

### GLP-1 and PYY secretion

STC-1 cells were washed three times with DMEM and incubated for 30 min at 37°C in DMEM containing various test reagents. After incubation, the conditioned medium was collected and the concentration of GLP and PYY was determined by enzyme immunoassay with a specific ELISA kits for GLP-1 and PYY (Peninsula Laboratories, LLC, San Carlos, CA) (Katsuma et al., 2005).

### Assay of phosphoinositide (PI) hydrolysis

PI hydrolysis was measured as total inositol phosphate formation with the use of anion exchange chromatography as described previously (Murthy and Makhlouf, 1996). Cells were labeled with myo-2-[^3^H]inositol (0.7 Ci/ml) in inositol-free medium and treated with OA or 8-pCPT-2′-*O*-Me-cAMP (10 μM) in the presence or absence of inhibitors for 1 min in a medium containing 25 mM HEPES, 115 mM NaCl, 5.8 mM KCl, 2.1 mM KH_2_PO_4_, 2 mM CaCl_2_, 0.6 mM MgCl_2_, and 14 mM glucose. The reaction was terminated by the addition of 940 μl of chloroform-methanol-HCl (50:100:2). After extraction with 340 μl of chloroform and 340 μl of H_2_O, the upper aqueous phase was applied to DOWEX AG-1 columns. [^3^H]inositol phosphates were then eluted, and the radioactivity was determined by liquid scintillation.

### Statistical analysis

The results are expressed as means ± s.e.m. of *n* experiments, and statistical significance was determined using Student's *t*-test for paired or unpaired values.

## Results

### Expression of TGR5, CSE, and CBS in STC-1 cells

**Figure 1:**
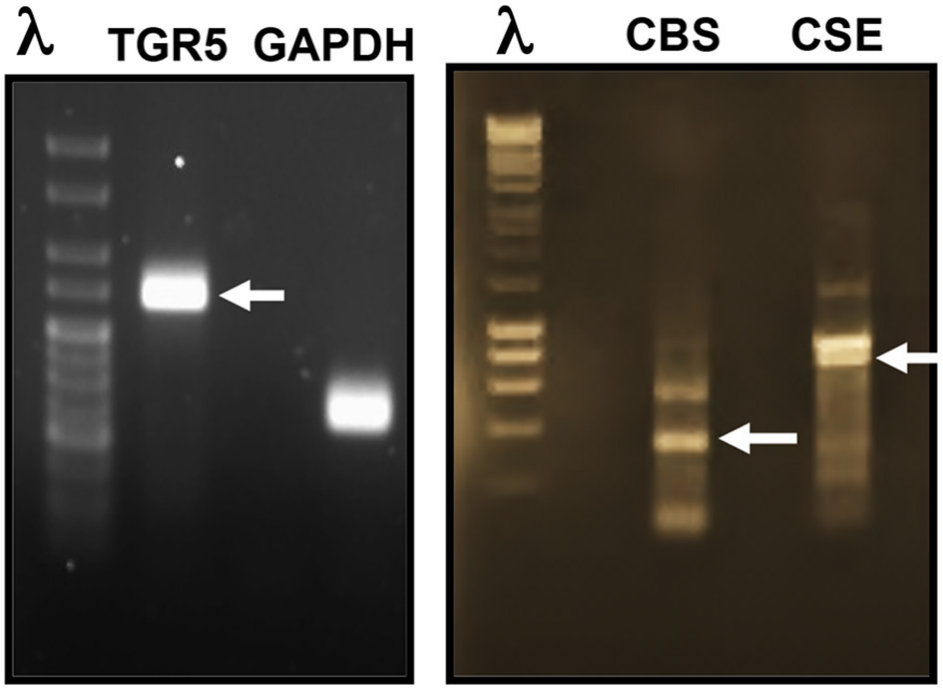
**Expression of TGR5, CBS, and CSE in STC-1 cells**. RT-PCR analysis of mRNA using specific primers showed amplification of PCR products of predicted size for TGR5 (269 bp), CBS (345 bp), and CSE (851 bp).

To gain insight into the role of TGR5 and H_2_S in the regulation of GLP-1 and PYY release, we first investigated the expression of TGR5 and the endogenous enzymes involved in the synthesis of H_2_S in STC-1 cells. RT-PCR analysis of mRNA using specific primers showed amplification of PCR products of predicted size for TGR5 (269 bp), CBS (345 bp), and CSE (851 bp) (Figure 1).

### OA mediated GLP-1 and PYY release via TGR5

**Figure 2:**
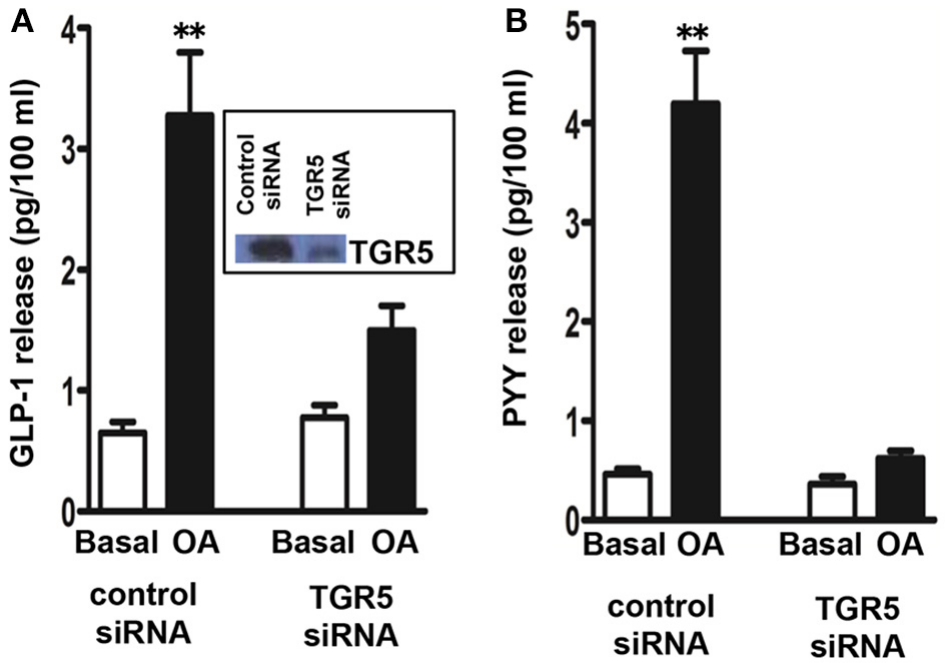
**TGR5-mediated release of GLP-1 and PYY by oleanolic acid (OA)**. Cells transfected with control siRNA or TGR5-specific siRNA for 48 h and then treated with OA (10 μM) for 30 min. Release of GLP-1 **(A)** and PYY **(B)** into the medium was measured by ELISA. *Inset:* Down regulation of TGR5 expression in cells transfected with TGR5 siRNA was determined by western blot. Results are expressed as pg/100 ml. Values are mean ± s.e.m. of 5 experiments. ^**^p < 0.001 vs. basal.

We initially examined whether STC-1 cells are responsive to the TGR5 selective ligand OA to release GLP-1 and PYY. The dose of OA was selected based on our previous work in pancreatic β cells and isolated smooth muscle cells (Kumar et al., 2012; Rajagopal et al., 2013). Treatment of STC-1 cells with OA (10 μM) stimulated GLP-1 and PYY release over a 30 min incubation period (4.1 ± 0.6-fold increase in GLP-1 release above basal levels 0.65 ± 0.09 pg/100 ml; 8.3 ± 1.2-fold increase in PYY release above basal levels 0.42 ± 0.068 pg/100 ml). The specific involvement of TGR5 in GLP-1 and PYY release in response to OA was examined using TGR5 specific siRNA. Neither basal nor OA-induced GLP-1 and PYY secretion was affected in cells transfected with control siRNA (Figure 2); however, the effect of OA on GLP-1 and PYY release was significantly attenuated in cells transfected with TGR5 siRNA compared to cells transfected with control siRNA (78 ± 10% inhibition in GLP-1 release and 88 ± 12% inhibition in PYY release) (Figure 2). Western blot analysis demonstrated suppression of TGR5 expression in cells transfected with TGR5 siRNA (Figure 2; *inset*). These results suggest that OA-induced GLP-1 and PYY release is mediated by TGR5, but not by other receptors such as nuclear receptor FXR.

### Signaling pathways activated by TGR5

#### Activation of GS/cAMP pathway

**Figure 3:**
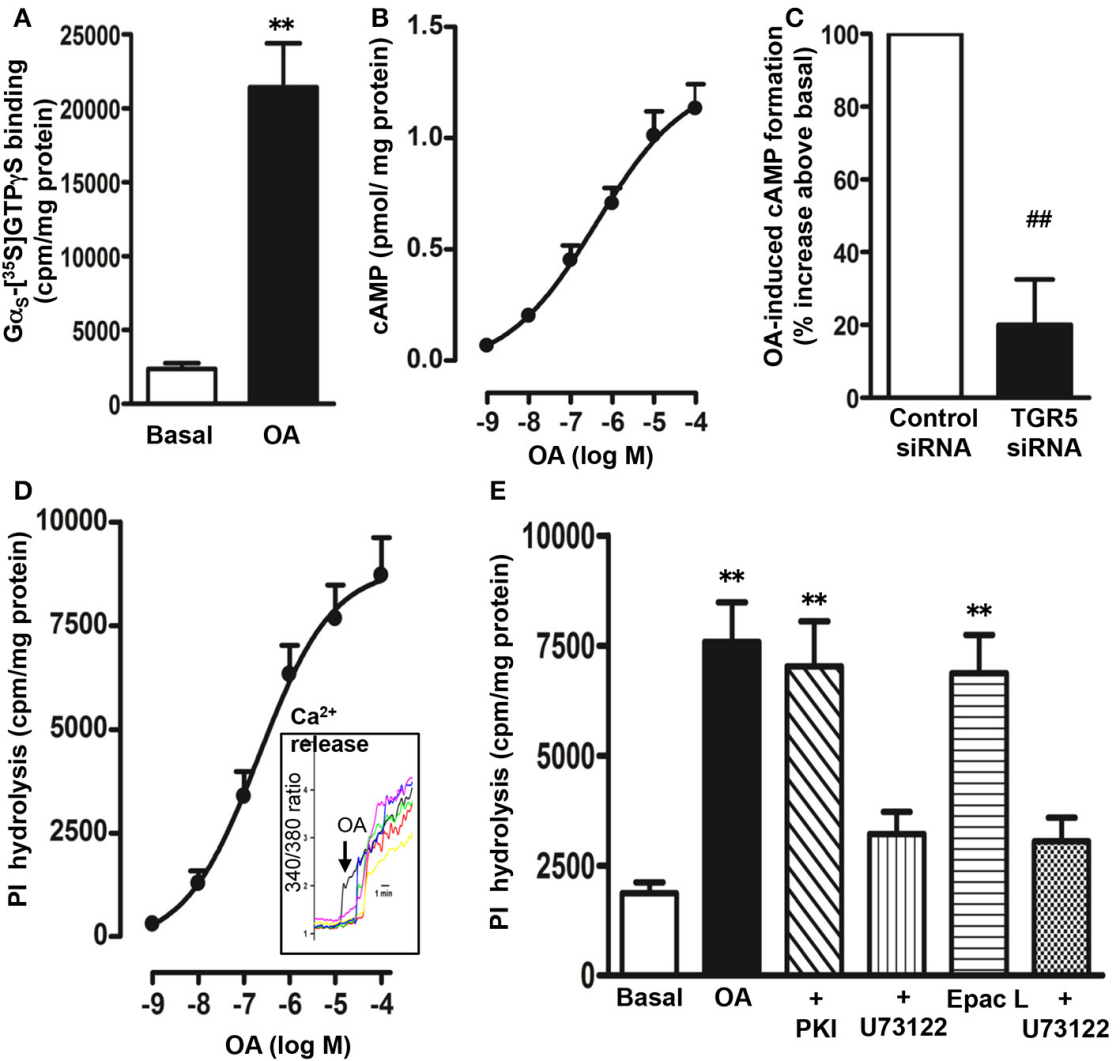
**Signaling pathways activated by OA**. **(A)** Selective activation of Gs by OA. Membranes were incubated with [^35^S]GTPγS in the presence or absence of OA (10 μM) for 20 min. Aliquots were added to wells pre-coated with antibodies to Gα_s_, Gα_q_, Gα_i1_, Gα_i2_, and Gα_i3_ for 2 h and the bound radioactivity was measured. An increase in the binding of [^35^S]GTPγS.Gα reflects activation of G protein. A significant increase in the binding of [^35^S]GTPγS.Gα complexes was obtained to wells coated with Gα_s_ antibody only. No significant increase in the binding was obtained to wells coated with Gα_i1_ (5 ± 7% increase), Gα_i2_ (3 ± 8 increase), Gα_i3_ (6 ± 9% increase), or Gα_q_ (10 ± 15% increase) antibody. Values are mean ± s.e.m. of 4 experiments. ^**^p < 0.001 vs. basal. **(B)** Stimulation of cAMP by OA. Cells were treated with different concentrations of OA for 5 min in the presence of IBMX (10 μM) and cAMP formation was measured by radioimmunoassay. Results are expressed as pmol/mg protein above basal levels (0.054 ± 0.008 pmol/mg protein). OA caused an increase in cAMP levels in a concentration-dependent manner. Values are mean ± s.e.m. of 3 experiments. **(C)** TGR5-mediated increase in cAMP by OA. Cells transfected with control siRNA or TGR5-specific siRNA were treated with OA (10 μM) for 5 min and cAMP formation was measured by radioimmunoassay. Suppression of TGR5 expression by TGR5 siRNA was confirmed by western blot analysis. Basal level of cAMP was similar in cells transfected with control siRNA (0.051 ± 0.007 pmol/mg protein) and or TGR5 siRNA (0.049 ± 0.008 pmol/mg protein). OA induced a significant increase in cAMP levels (1.12 ± 0.08 pmol/mg protein) in control cells, but not in cells transfected with TGR5 siRNA. Results are expressed as percent of response. Values are mean ± s.e.m. of 3 experiments. ^##^*p* < 0.001 significant inhibition of cAMP response compared to cells transfected with control siRNA. **(D,E)** Stimulation of PI hydrolysis and Ca^2+^ release by OA and 8-pCPT-2′-*O*-Me-cAMP (Epac ligand). Cells labeled with myo-[^3^H]inositol were treated with different concentrations of OA and PI hydrolysis was measured **(D)**. Cells were incubated with the cAMP analog that selectively activates Epac, 8-pCPT-2′-*O*-Me-cAMP (10 μM) or OA (10 μM) in the presence or absence of inhibitors of PI hydrolysis (U73122, 10 μM) or PKA (myristoylated PKI, 1 μM) **(E)**. PI hydrolysis was measured by ion exchange chromatography as increase in water soluble inositol formation. Results are expressed as cpm/mg protein above basal levels (1856 ± 302 cpm/mg protein). Treatment of cells with PKI (1985 ± 402 cpm/mg protein) or U73122 (1685 ± 293 cpm/mg protein) alone had no significant effect on basal PI hydrolysis. Values are mean ± s.e.m. of 4 experiments. ^**^p < 0.001 vs. basal. **(D)** *Inset*: Increase in intracellular Ca^2+^ monitored in cells after loading the monolayers with Fura-2 AM. Increase in Ca^2+^ is represented in the figure as increase in 340/380 ratio.

In membranes isolated from STC-1 cells, OA (10 μM) caused a significant increase in the binding of [^35^S]GTPγS to Gα_s_ as determined by the binding of [^35^S]GTPγS.Gα complexes to the corresponding Gα_s_ antibody (10 ± 2-fold increase in the binding) (Figure 3A). There was no significant increase in the binding of [^35^S]GTPγS to Gα_i1_ (5 ± 7% increase), Gα_i2_ (3 ± 8% increase), Gα_i3_ (6 ± 9% increase), or Gα_q_ (10 ± 15% increase) antibody. These results suggest that TGR5 is selectively coupled to the activation of G_s_. Consistent with activation of Gα_s_, OA caused an increase in cAMP formation in a concentration-dependent manner with an EC_50_ of 0.7 ± 0.1 μM and a maximum stimulation of 1.20 ± 0.07 pmol/mg protein above basal levels (0.054 ± 0.008 pmol/mg protein) was obtained with 10 μM OA (Figure 3B).

The specific involvement of TGR5 in the cAMP response to OA was further examined using TGR5 specific siRNA. Neither basal nor OA-induced cAMP formation was affected in cells transfected with control siRNA (basal, 0.051 ± 0.007 pmol/mg protein; OA, 1.12 ± 0.08 pmol/mg protein); however, the effect of OA on cAMP formation was significantly attenuated in cells transfected with TGR5 siRNA (81 ± 6% inhibition) compared to control cells (Figure 3C).

#### Stimulation of PI hydrolysis and Ca^2+^ release

OA selectively activated Gα_s_, and treatment of myo-[^3^H]inositol labeled cells with OA resulted in an increase in PI hydrolysis in a concentration-dependent manner with an EC_50_ of 0.83 ± 0.15 μM and a maximal stimulation of 8715 ± 1024 cpm/mg protein above basal levels (1856 ± 302 cpm/mg protein) with 10 μM OA (Figure 3D). OA-induced PI hydrolysis was blocked (81 ± 7% inhibition) by a selective PI hydrolysis inhibitor, U73122 (10 μM), but not by a selective PKA inhibitor, myristoylated PKI (1 μM) (8 ± 5% inhibition) (Figure 3E). These results combined with selective activation of Gα_s_ by OA suggest that stimulation of PI hydrolysis is independent of PKA, and probably involves activation of the cAMP-dependent Epac/PLC-ε pathway. In support to this notion, RT-PCR studies demonstrated expression of both Epac2 and PLC-ε in STC-1 cells (data not shown) and a selective Epac ligand, 8-pCPT-2′-*O*-Me-cAMP (10 μM) stimulated PI hydrolysis that was significantly inhibited (75 ± 5% inhibition) by U73122 (1 μM) (Figure 3E). Stimulation of PI hydrolysis by OA was corroborated by Ca^2+^ release studies. Increase in intracellular Ca^2+^ was monitored in cells after loading the monolayers with fura-2 AM. Increase in cytosolic Ca^2+^ was measured as increase in 340/380 ratio. OA caused a rapid increase in Ca^2+^ response with a mean increase of 4.1 ± 0.7-fold (*n* = 25 cells, *p* < 0.001) above base line (Figure 3D; *inset*).

**Figure 4:**
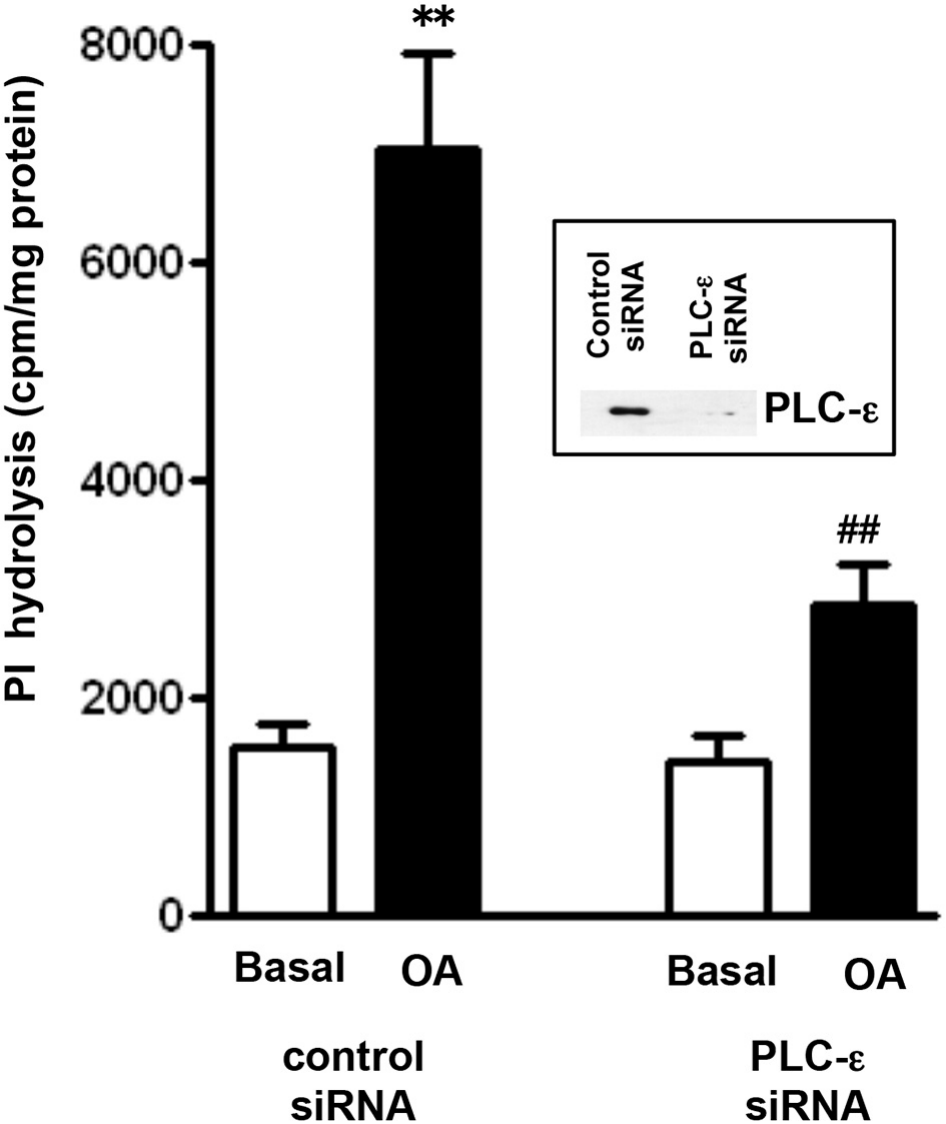
**OA-induced PI hydrolysis is mediated via PLC-ε**. Cells transfected with PLC-ε specific siRNA or control siRNA were labeled with myo-[^3^H]inositol. Cells were treated with OA (10 μM) for 60 s and PI hydrolysis was measured by ion exchange chromatography as increase in water soluble inositol formation. Results are expressed as cpm/mg protein. Values are mean ± s.e.m. of 4 experiments. ^**^p < 0.001 vs. Basal ^##^*p* < 0.001 significant inhibition of PI hydrolysis compared to response in cells transfected with control siRNA. *Inset*: Expression of PLC-ε in control cells and in cell transfected with PLC-ε siRNA.

The involvement of PLC-ε in OA-stimulated PI hydrolysis was examined using PLC-ε siRNA. Suppression of PLC-ε expression by transfection of PLC-ε siRNA was validated by western blot analysis (Figure 4). Neither basal nor OA-induced PI hydrolysis was affected in cells transfected with control siRNA. Stimulation of PI hydrolysis in response to OA (10 μM), however, was significantly inhibited in cells transfected with PLC-ε siRNA (72 ± 5% inhibition) (Figure 4).

**Figure 5:**
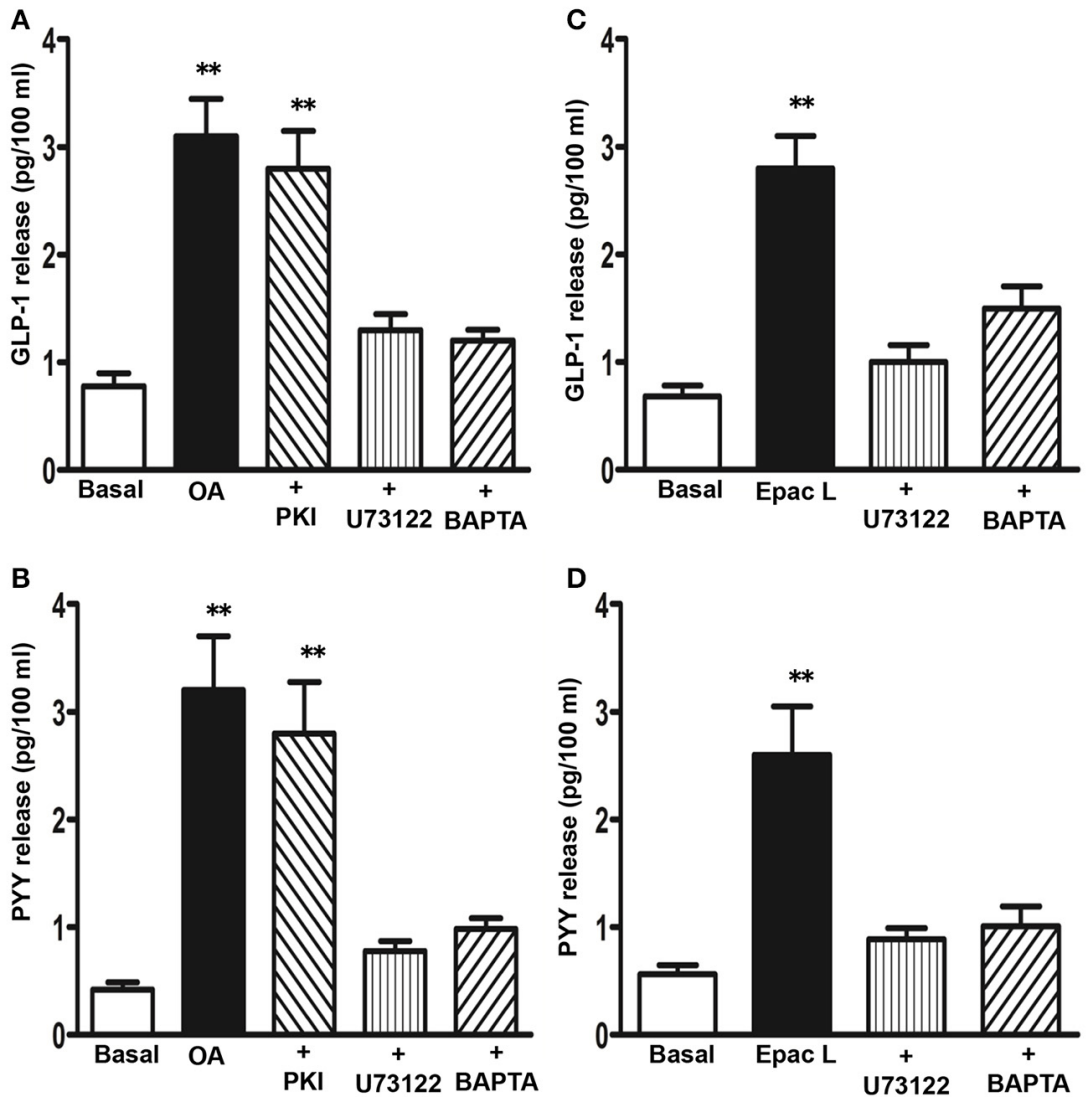
**Signaling pathways involved in the release of GLP-1 and PYY by OA**. Cells were treated with OA (10 μM) **(A,B)** or 8-pCPT-2′-*O*-Me-cAMP (Epac L, 10 μM) **(C,D)** for 30 min in the presence or absence of inhibitors of PI hydrolysis (U73122, 10 μM), PKA (myristoylated PKI, 1 μM), or Ca^2+^ chelator (BAPTA-AM, 10 μM). Release of GLP-1 and PYY into the medium was measured by ELISA and the results are expressed as pg/100 ml. Treatment of cells with PKI, U73122 or BAPTA-AM alone had no significant effect on basal release. Values are mean ± s.e.m. of 6 experiments. ^**^p < 0.001 vs. basal.

The involvement of PI hydrolysis/Ca^2+^ and cAMP/PKA pathways in OA-mediated GLP-1 and PYY release was examined using selective inhibitors. OA-induced GLP-1 release was significantly inhibited by incubation of cells with U73122 or with the Ca^2+^ chelator, BAPTA-AM (78 ± 6 and 82 ± 5% inhibition, respectively), but not with myristoylated PKI (14 ± 8% inhibition) (Figure 5A). Similarly, OA-induced PYY release was inhibited by U73122 or BAPTA-AM (86 ± 8 and 79 ± 6% inhibition, respectively), but not myristoylated PKI (12 ± 6% inhibition) (Figure 5B). 8-pCPT-2′-*O*-Me-cAMP also caused an increase in GLP-1 (3.8 ± 0.5-fold increase) and PYY release (4.2 ± 0.7-fold increase) that was inhibited by U73122 (84 ± 7% inhibition in GLP-1 release and 65 ± 6% inhibition in PYY release) or BAPTA-AM (83 ± 6% inhibition in GLP-1 release and 78 ± 5% inhibition in PYY release) (Figures 5C,D). These results suggest that TGR5 activation causes GLP-1 and PYY release via the cAMP/Epac/PLC-ε pathway. A similar pathway was shown to be involved in the release of insulin in response to TGR5 ligands in pancreatic β cells (Kumar et al., 2012).

#### Inhibition of OA-induced GLP-1 and PYY release by H_2_S

**Figure 6:**
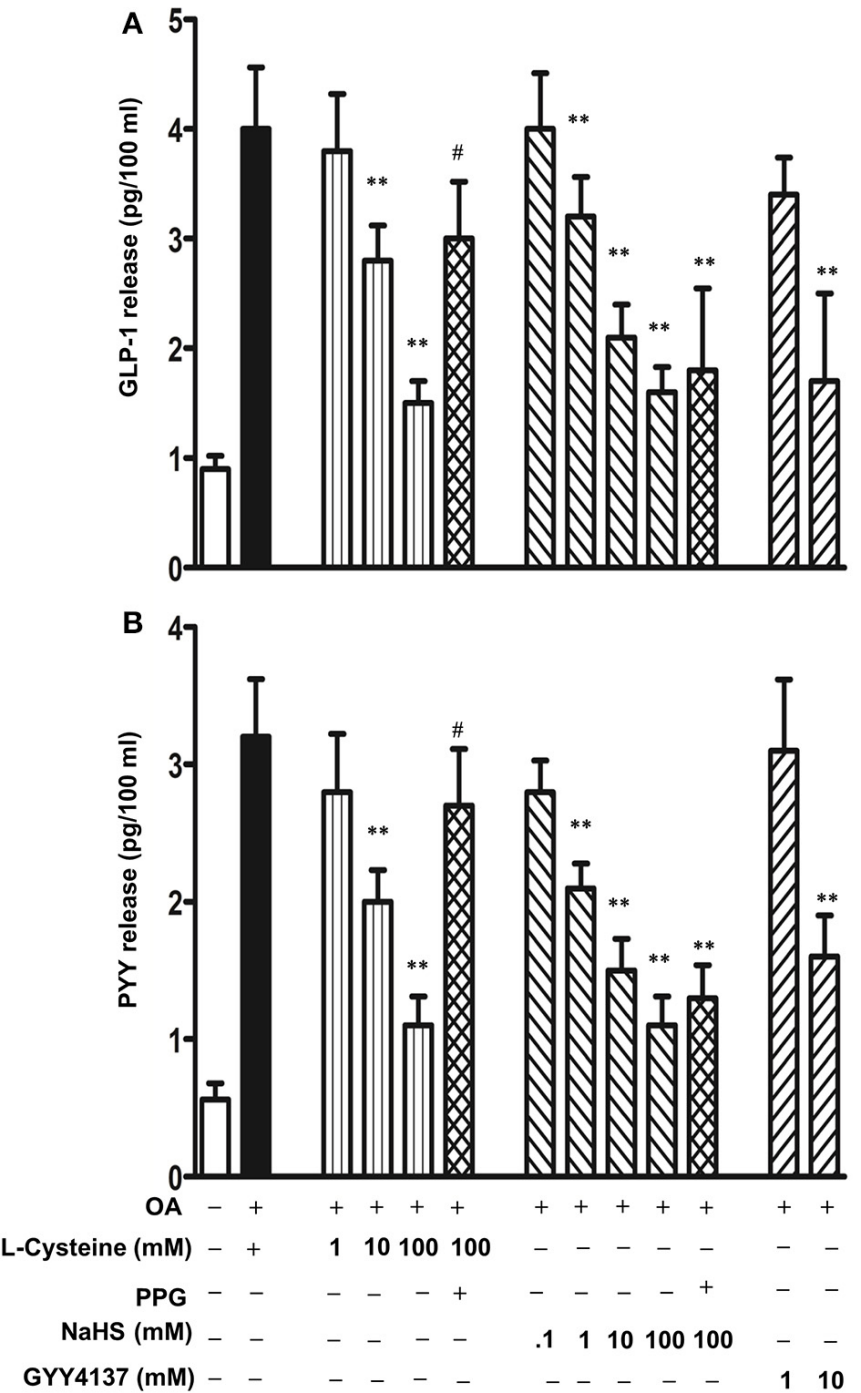
**Inhibition of OA-induced GLP-1 and PYY release by H_2_S**. Cells were incubated with OA (10 μM) in the presence or absence of various concentrations of L-cysteine, an activator of endogenous CSE and CBS, and H_2_S donors NaHS or GYY4137. In some experiments, L-cysteine (100 mM) was co-incubated with the CSE inhibitor, DL-propargylglycine (PPG). GLP-1 **(A)** and PYY **(B)** release into the medium was measured by ELISA and the results are expressed as pg/100 ml. Values are mean ± s.e.m. of 4 experiments. ^**^p < 0.01 vs. response to OA alone; ^#^*p* < 0.05 vs. OA+L-cysteine (100 mM).

To investigate the regulation of TGR5-mediated GLP-1 and PYY release by H_2_S, cells were incubated with L-cysteine, an activator of endogenous enzymes CSE and CBS, for 10 min and then GLP-1 and PYY release in response to OA was measured. L-cysteine inhibited both GLP-1 and PYY release in a dose-dependent manner (Figures 6A,B). Maximum inhibition of GLP-1 and PYY release by L-cysteine is closely similar (78 ± 7% inhibition in GLP-1 release and 82 ± 8% inhibition in PYY release). To further examine, whether endogenous H_2_S production was involved in the inhibition by L-cysteine, DL-propargylglycine (PPG), a selective inhibitor of CSE was used (Yang et al., 2005). PPG pretreatment significantly reversed the inhibition of OA-induced GLP-1 and PYY release by L-cysteine (25 ± 4% inhibition in GLP-1 release and 25 ± 4% inhibition in PYY release) (Figures 6A,B). Treatment of cells with NaHS, an exogenous donor of H_2_S, also inhibited both GLP-1 and PYY release in a concentration-dependent manner. Maximum inhibition of GLP-1 and PYY release by NaHS was similar (78 ± 8% inhibition in GLP-1 release and 79 ± 6% inhibition in PYY release) (Figures 6A,B). PPG had no effect on NaHS (100 mM)-induced inhibition of GLP-1 (74 ± 5% inhibition) or PYY release (71 ± 3% inhibition) (Figures 6A,B). Treatment of cells with GYY4137, an exogenous donor that releases H_2_S with slower kinetics, also inhibited both GLP-1 and PYY release (43 ± 4% inhibition in GLP-1 release and 68 ± 6% inhibition in PYY release with 10 mM GYY4137) (Figures 6A,B).

#### Effect of H_2_S on G_s_/cAMP and Epac/PLC-ε pathways

**Figure 7:**
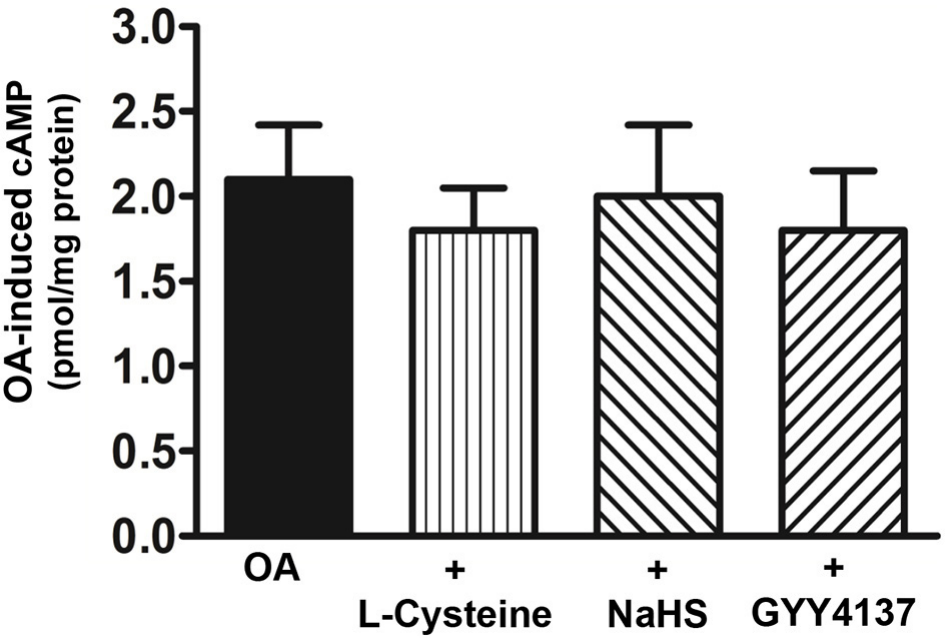
**Lack of effect of H_2_S on OA-induced cAMP formation**. Cells were incubated with OA (10 μM) in the presence or absence of L-cysteine (100 mM), NaHS (10 mM), or GYY4137 (10 mM) for 5 min. cAMP formation was measured by radioimmunoassay. Results are expressed as pmol/mg protein above basal levels (0.054 ± 0.008 pmol/mg protein). Values are mean ± s.e.m. of 5 experiments.

**Figure 8:**
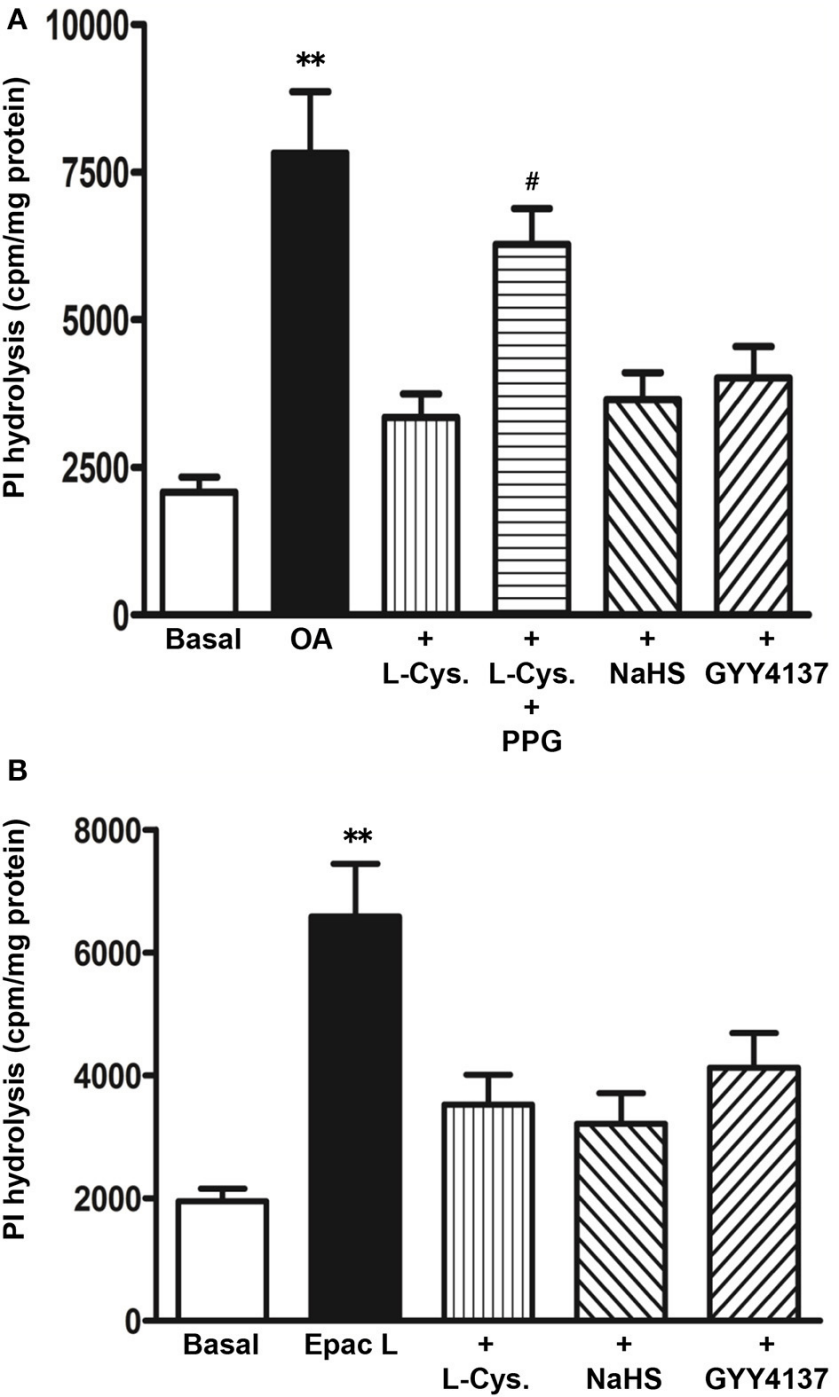
**Inhibition of OA-induced PI hydrolysis by H_2_S. (A)** Myo[^3^H]inositol labeled cells were incubated with OA (10 μM) in the presence or absence of L-cysteine (100 mM), NaHS (10 mM), or GYY4137 (10 mM). In some experiments L-cysteine was co-incubated with the CSE inhibitor, DL-propargylglycine (PPG). **(B)** Myo[^3^H]inositol labeled cells were incubated with 8-pCPT-2′-*O*-Me-cAMP (Epac L, 10 μM) in the presence or absence of L-cysteine (100 mM), NaHS (10 mM), or GYY4137 (10 mM). PI hydrolysis was measured by ion-exchange chromatography as increase in water soluble inositol formation. Results are expressed as cpm/mg protein. Values are mean ± s.e.m. of 3 experiments. ^**^p < 0.001 vs. basal; ^#^*p* < 0.05 vs. OA+L-cysteine.

To examine whether the G_s_/cAMP pathway was inhibited by H_2_S, cAMP formation in response to TGR5 activation with OA was measured in the presence of L-cysteine, NaHS or GYY4137. cAMP levels were increased in response to OA (2.1 ± 0.3 pmol/mg protein increase above basal 0.093 ± 0.01 pmol/mg protein) and the increase was not significantly affected by L-cysteine, NaHS or GYY4137 (Figure 7). These results raised the possibility that the inhibition of OA-induced GLP-1 and PYY release by L-cysteine or H_2_S donors could be due to inhibition of targets downstream of G_s_/cAMP. We tested the possibility that inhibition of OA-induced peptide release by L-cysteine and H_2_S donors is due to inhibition of OA-induced PI hydrolysis. In contrast to its effect on OA-induced cAMP formation, L-cysteine inhibited OA-induced PI hydrolysis (81 ± 8% inhibition) and the inhibition was significantly reversed by PPG (28 ± 4% inhibition) (Figure 8A). OA-induced PI hydrolysis was also inhibited by NaHS (72 ± 5% inhibition) or GYY4137 (63 ± 6% inhibition) (Figure 8A). Stimulation of PI hydrolysis, independent of TGR5 activation, by 8-pCPT-2′-*O*-Me-cAMP was also inhibited by L-cysteine (66 ± 5% inhibition), NaHS (72 ± 7% inhibition), or GYY4137 (53 ± 3% inhibition) (Figure 8B). These results suggest that inhibition of OA-induced GLP-1 and PYY release by L-cysteine, NaHS or GYY4137 was mediated via inhibition of Epac/PLC-ε pathway.

## Discussion

Our studies demonstrate that activation of TGR5 receptors in enteroendocrine cells leads to activation of the Gα_s_/cAMP/Epac/PLC-ε/Ca^2+^ pathway and stimulation of GLP-1 and PYY release. These conclusions are reached through several lines of evidence. (1) Specific involvement of TGR5 in GLP-1 and PYY release is supported by the finding that suppression of TGR5 by siRNA greatly attenuated the increase in cAMP formation and GLP-1 and PYY release in response to OA. (2) Selective activation of G_s_ and stimulation of adenylyl cyclase activity and PI hydrolysis, and Ca^2+^ release occurs in response to OA. (3) OA-induced PI hydrolysis and GLP-1 and PYY release was blocked by suppression of PLC-ε and PI hydrolysis inhibitor (U73122), but not by a PKA inhibitor (myristoylated PKI). (4) Suppression of PLC-ε expression blocked the increase in PI hydrolysis in response to OA. These data, in conjunction with the data using a cAMP analog that selectively activated Epac and which also caused stimulation of PI hydrolysis and GLP-1 and PYY release, provided further confirmation of the key role of the Epac/PI-PLC/Ca^2+^ pathway in GLP-1 and PYY release. It is noteworthy that in the present study we used OA, a selective agonist of the TGR5 receptor, rather than native bile salts, to examine the regulation of PYY and GLP-1 release (Sato et al., 2007). Native Bile salts activate two receptors, the nuclear FXR receptor, and the G-protein coupled TGR5 receptor. The former likely mediates more delayed genomic actions of bile salts while the latter likely mediates more immediate or rapid actions of bile salts. The EC_50_ for TGR5 ligand stimulated cAMP formation and PI hydrolysis is close to the luminal bile acids concentrations and suggests that under physiological conditions it is likely that L cells would be activated by postprandial bile acids and induce GLP-1 and PYY release.

Previous studies have established that bile acids stimulate the release of GLP-1 from enteroendocrine cells, both *in vivo* and *in vitro* (Katsuma et al., 2005; Parker et al., 2012). TGR5 reportedly couples to the Gs/cAMP pathway to mediate GLP-1 release from enteroendocrine cells (Katsuma et al., 2005; Parker et al., 2012). These studies, however, did not examine the pathway in detail and presumed that cAMP acted through its canonical kinase, PKA, to cause the release of GLP-1. Our studies provided a more detailed analysis of the signaling pathways and demonstrate that release of GLP-1 and PYY was mediated via a PKA-independent cAMP/Epac/PLC-ε/Ca^2+^ pathway. A similar PKA-independent pathway was recently demonstrated in TGR5-mediated insulin release in pancreatic β cells (Kumar et al., 2012). The increased cytosolic Ca^2+^ triggers fusion of peptide-containing secretory granules with the plasma membrane and stimulates secretion from the cells. Although, the present study did not provide evidence for the involvement of Epac in OA- mediated PI hydrolysis, demonstration of Epac2 expression in STC-1 cells and published reports in other cell types support our conclusion about the role of Epac/PLC-ε pathway in Ca^2+^ mobilization. Epac is a cAMP-regulated guanine nucleotide exchange factor (GEF) and regulates many physiological functions via activation of monomeric G protein Rap1 (De Rooij et al., 1998). Binding of cAMP to Epac stimulates its GEF activity and activates Rap1. Rap1 is active in the GTP-bound form and possesses intrinsic GTPase activity that is activated by GTPase activating protein, Rap1GAP. Epac2 has been shown to activate PLC-ε via Rap1. Deletion of PLC-ε or Epac2 gene *in vivo* or expressing Rap1GAP that down regulates Rap1 activity *in vitro* attenuates the effect of 8-pCPT-2′-*O*-Me-cAMP on Ca^2+^ release and insulin release (Dzhura et al., 2011). The relative importance of Epac2 and Rap1 in TGR5 mediated GLP-1 and PYY release remains to be determined.

Recent studies have demonstrated the expression of TGR5 in various cell types of the gastrointestinal tract (Lavoie et al., 2010; Alemi et al., 2013) and we have confirmed the presence of TGR5 receptors on STC-1 cells by the presence of specific mRNA. Our studies coupled with the demonstration of TGR5 receptors on the native enteroendocrine cells, strongly suggest that bile acids in the gut can directly influence enteroendocrine cells to increase the release of GLP-1 and PYY, and regulate energy metabolism and gastrointestinal motility. Activation of TGR5 could regulate motility via several mechanisms including release of 5-HT from enteroendocrine cells and initiation of peristalsis, activation of nitrergic neurons and suppression of spontaneous contraction, and direct activation of smooth muscle cells to mediate muscle relaxation (Alemi et al., 2013). In addition to these direct effects mediated by TGR5 receptors on target cells, bile salts are likely to have indirect effects on gut motility. Both GLP-1 and PYY are shown to regulate gastrointestinal functions, especially proximal gut motility and secretion (Grudell and Camilleri, 2007; Holst, 2007; Kidd et al., 2008; Hellstrom, 2010; Holzer et al., 2012). The biological functions of PYY include attenuation of food intake, and gastric emptying and motility, and gastric secretion. PYY modulates gastrointestinal functions and motility partly via its action on myenteric neurons and smooth muscle but also through central effects mediated by the vagus (Grudell and Camilleri, 2007; Holzer et al., 2012). This latter mechanism is especially noteworthy in the postprandial state where PYY has been shown to participate in the inhibition of gastric motility and secretion when intraluminal stimuli reach the distal gut, a phenomenon known as the ileal brake (Grudell and Camilleri, 2007; Holzer et al., 2012). The presence of TGR5 receptors on enteroendocrine cells mediating the release of PYY and the presence of bile salts in the lumen of the distal gut suggest that TGR5 receptor activation may play a major role in initiating the ileal break (Al-Saffar et al., 1985; Savage et al., 1987; Pironi et al., 1993; Goumain et al., 1998; Misra et al., 2004; Murphy and Bloom, 2006).

The postprandial functions of GLP-1 include insulin release from pancreatic β cells and regulation of glucose metabolism (Toft-Nielsen et al., 2001). Studies using pharmacological agents and transgenic animal models established the physiological functions of TGR5 and GLP-1 secretion. The impact of GLP-1 mimetic agents and TGR5 agonists to stimulate GLP-1 release in the diabetes, illustrates the beneficial effects of GLP-1 in the regulation of pancreatic β cell function. Increased delivery of bile acids to distal gut following gastric bypass, and hence their increased stimulation of TGR5 receptors present on enteroendocrine cells in the distal gut, are correlated with the elevated levels of GLP-1 and improvement of glucose tolerance (Hage et al., 2012). In addition, recent studies demonstrated that GLP-1 functions as a trophic factor for islet cells and augments glucose-dependent insulin secretion (Buteau et al., 2003). Our recent studies demonstrated the TGR5 receptors are also present in pancreatic β cells and that activation of these receptors by bile acids results in insulin secretion (Kumar et al., 2012). These multiple functions of TGR5 act in concert and offer a great potential for the treatment of hyperglycemia.

Other important results of the present study demonstrate that TGR5-induced release of GLP-1 and PYY from enteroendocrine cells is modulated by endogenous agents produced within the wall of the gut such as H_2_S (Linden et al., 2010; Strege et al., 2011; Vandiver and Snyder, 2012). In support to this notion, we have shown: (i) L-cysteine, an activator of H_2_S producing enzymes (CSE and CBS), or the exogenous H_2_S donors NaHS and GYY4137 inhibited OA-induced GLP-1 and PYY release, and (ii) inhibition of release is due to suppression of the Epac/PLC-ε/Ca^2+^ pathway downstream of cAMP pathway. Our results also demonstrated that when CSE was inhibited by the specific inhibitor DL-propargylglycine (PPG), inhibition of GLP-1 and PYY release was greatly attenuated suggesting a role for CSE in endogenous H_2_S generation and of H_2_S in regulating the TGR5-mediated release of GLP-1 and PYY. Similar levels of inhibition of OA-induced GLP-1 and PYY release with the rapid releasing donor, NaHS, and the slow releasing donor, GYY4137, suggest that the rate of H_2_S generation is not an important factor in the inhibitory action. The contractile effect of oxytocin in rat and human myometrium was also inhibited by both NaHS and GYY4137 in the same manner (Robinson and Wray, 2012). This is in contrast to the divergent actions of NaHS and GYY4137 on LPS-induced release of pro-inflammatory mediators. GYY4137 inhibited pro-inflammatory mediators, whereas NaHS stimulated pro-inflammatory mediators (Whiteman et al., 2010; Wallace et al., 2012). NaHS results in rapid release of a large bolus of H_2_S which could be toxic and is not physiological. The slow release of H_2_S generated by GYY4137, is certainly more physiological in time course, and is comparable to the effect of endogenous H_2_S generated from CSE and CBS activity (Robinson and Wray, 2012). The effect of H_2_S on insulin secretion and smooth muscle contraction was demonstrated largely to be due to the opening of K_ATP_ channels (Yang et al., 2005; Mustafa et al., 2009; Jiang et al., 2010; Tang et al., 2010; Wallace et al., 2012; Wang, 2012). The role of K_ATP_ channels in mediating the effects of H_2_S on GLP-1 and PYY release induced by activation of TGR5 receptors was not evident in the present study. In contrast this study indicates that H_2_S acts downstream of the cAMP generation induced by TGR5 activation, most likely by inhibiting PI hydrolysis and the subsequent increase in intracellular calcium needed for vesicle release from the enteroendocrine cells.

**Figure 9:**
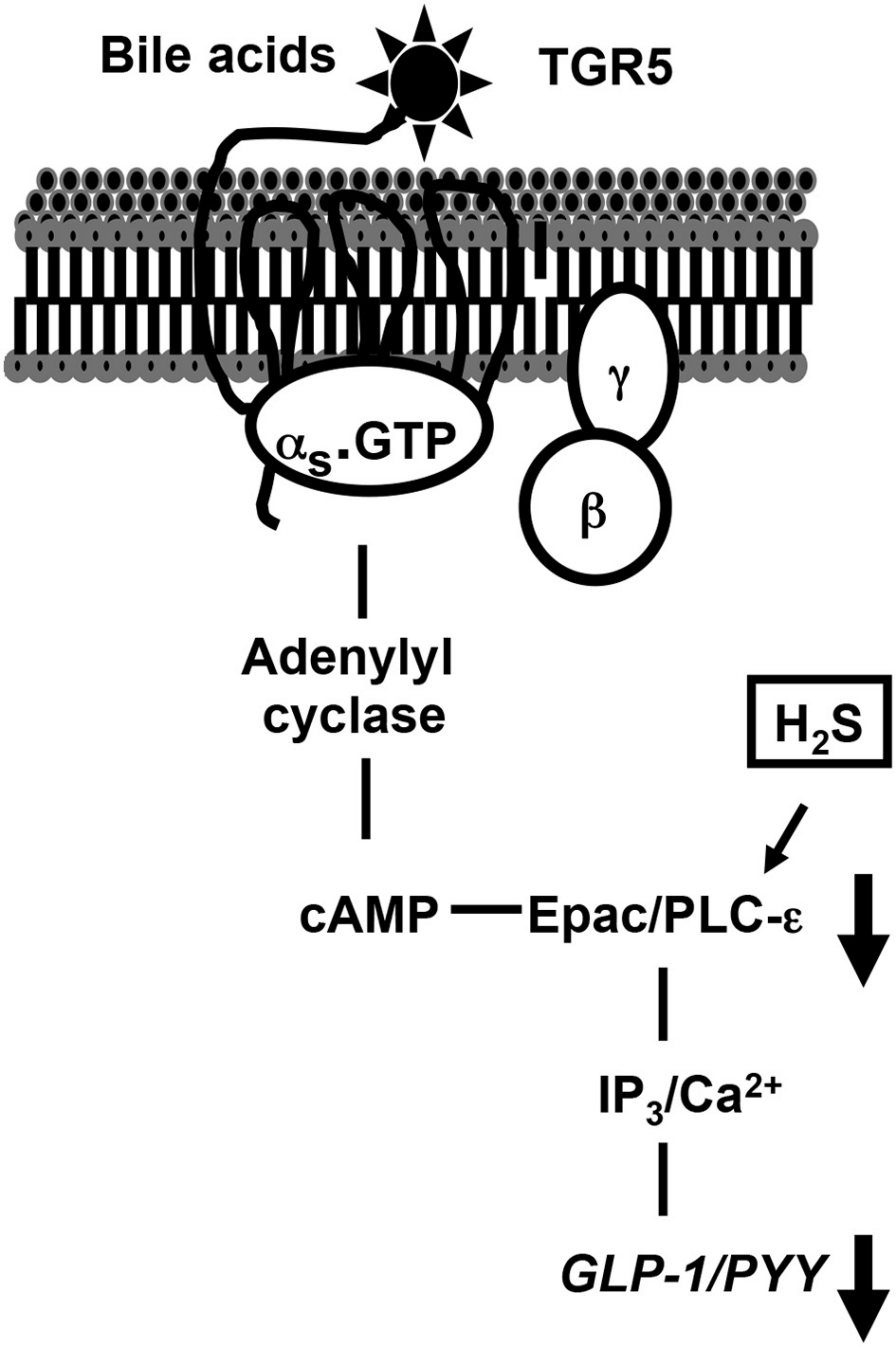
**TGR5-mediated signaling pathways to release GLP-1 and PYY and inhibition of release of GLP-1 and PYY by H_2_S**. In STC-1 cells, activation of G_s_-coupled TGR5 receptors by OA causes stimulation of PI hydrolysis, and release of GLP-1 and PYY via a PKA-independent, cAMP-dependent mechanism involving Epac/PLC-ε/Ca^2+^ pathway. H_2_S inhibits GLP-1 and PYY release by inhibiting PI hydrolysis and Ca^2+^ release.

In conclusion, we have provided evidence indicating that GLP-1 and PYY release in response to TGR5 activation is mediated via the Gs/cAMP/PLC/Ca^2+^ pathway and both endogenous and exogenous H_2_S inhibit TGR5-mediated GLP-1 and PYY release. Inhibition of TGR5 mediated effects by H_2_S is due to inhibition of PI hydrolysis (Figure 9). Sulfur-reducing bacteria, present as a part of the microbiota of the gut, generate H_2_S in the lumen where it has immediate access to enteroendocrine cells. The endogenous generation of H_2_S by CBS and CSE in mammalian cells in the gut wall such as smooth muscle cells in the adjacent muscle likely plays an important role in the regulation of gastrointestinal functions under physiological and pathophysiological conditions. To understand the physiological and pathophysiological significance of H_2_S, further studies are warranted to elucidate the mechanisms involved in the activation of endogenous H_2_S generating enzymes and identify the cellular targets of H_2_S which regulate GLP-1 and PYY release.

### Conflict of interest statement

The authors declare that the research was conducted in the absence of any commercial or financial relationships that could be construed as a potential conflict of interest.
